# Less Typical Courses of *Rhodococcus equi* Infections in Foals

**DOI:** 10.3390/vetsci9110605

**Published:** 2022-10-31

**Authors:** Alicja Rakowska, Agnieszka Marciniak-Karcz, Andrzej Bereznowski, Anna Cywińska, Monika Żychska, Lucjan Witkowski

**Affiliations:** 1 Division of Veterinary Epidemiology and Economics, Institute of Veterinary Medicine, Warsaw University of Life Sciences, 02-787 Warsaw, Poland; 2 Private Veterinary Practice, 13-400 Aubagne, France; 3 Faculty of Biological and Veterinary Sciences, Nicolaus Copernicus University, 87-100 Toruń, Poland

**Keywords:** *Rhodococcus equi*, extrapulmonary disorders, macrolide-induced anhidrosis, primary IgM deficiency

## Abstract

**Simple Summary:**

For decades, *Rhodococcus equi* infections remain one of the most common causes of death in foals before weaning, coming from endemic studs. The pathogen can create many problems other than its classical form—pneumonia. Such issues are described in the clinical cases presented in this paper. The first case concerns the potential contribution of rhodococcal infection to a grave outcome in a prematurely born foal lost as a yearling. Another presents so-called “extrapulmonary disorders” (EPD) and a theory of inherited immunodeficiency in a breeding dam line from one stud. The third case is connected with a suspected atypical location of the pulmonary abscess. The last example is associated with drug-induced anhidrosis. These clinical pictures may be considered rare or interesting and worth presenting in a scientific report.

**Abstract:**

This article aims to present several interesting and less typical courses of *Rhodococcus equi* infections in foals, collected during the 2019–2021 foaling seasons in some Polish studs. The study was conducted by the Division of Veterinary Epidemiology and Economics, Warsaw University of Life Sciences—SGGW, and concentrated on ultrasonographic contribution to diagnostics and treatment of the disease. Among many standard cases of rhodococcal pneumonia, some rare ones occurred. The aforementioned issues include the potential contribution of rhodococcal infection to a grave outcome in a prematurely born filly, lost as a yearling, so-called “extrapulmonary disorders” (EPD), a hypothesis of inherited immunodeficiency with grave outcome in a breeding dam line from one stud, and macrolide-induced anhidrosis. The main benefit of this report would be to supplement the general picture of clinical rhodococcosis.

## 1. Introduction

For many decades, *Rhodococcus equi* remains one of the main problems in foals before weaning worldwide. Despite being thoroughly described, this ubiquitous, opportunistic, intracellular, Gram + pathogen creates a severe risk of significant health problems and loss of foals in certain endemic studs. Its impact in a particular season and stud varies [1,2,3,4,5].

Commonly, rhodococcal infections are classified as pyogranulomatous pneumonia; however, the latest clinical papers identify many different forms of the disease, affecting tissues and organs other than the respiratory system. Increasing numbers of so-called “extrapulmonary changes” are being reported [4,6,7,8,9]. Additionally, many problems arise from the management of the disease. Lack of satisfying immunoprophylaxis, different approaches to diagnosis, no validated treatment alternatives, and problems with antimicrobial resistance creates a constant challenge for both researchers and field veterinarians [1,3,4,10,11,12,13,14].

This clinical report presents some less typical cases that have been presented for clinical and ultrasound evaluation during the studies on equine rhodococcosis provided in central and eastern Poland by the Division of Veterinary Epidemiology and Economics, Warsaw University of Life Sciences (SGGW), during 2019–2021 breeding seasons.

## 2. Clinical Cases: History and Examination Results

### 2.1. Clinical Approach

The monitoring program of rhodococcosis in foals including lung ultrasound was established in 5 horse studs in cooperation with SGGW. The participating studs are considered endemic based on the history of multiple *Rhodococcus equi* infections confirmed by post-mortem examinations and positive microbiological culture on blood agar, CAZ-NB and API Coryne tests from the samples collected in previous breeding seasons. A total of 192 foals were examined during the program. The screening aimed to improve and promote non-invasive and widely available field management of rhodococcosis in equine breeding centres. Considering the prior history of multiple foal loss due to *Rhodococcus equi* and scientific reports which claimed a higher probability of such infections in pneumonic foals of 2–3 months of age than any other pathogens [15,16,17,18,19], the diagnosis was based on the clinical picture and ultrasonographic evaluation of the lungs and pleural cavity (Draminski 4Vet Slim, linear transducer 8–10 MHz) [6,20,21]. Imaging diagnostics were performed roughly in two-week intervals, between the 3rd week and 4th month of life or later in case of clinical problems in older foals. The alluded approach emerged from a constant need to improve clinical diagnostics and treatment protocols which vary significantly in different seasons, counties, and studs [1,17,22,23].

#### 2.1.1. Case 1—Premature Foal

A one-month-old Anglo-Arabian filly, born nearly a month prematurely was appointed for a clinical and ultrasound examination in early June 2019. At the delivery, apparent signs of immaturity were observed, such as small size, domed forehead, silky hair, tendon laxity, and general weakness. Because of the presence of the suckling reflex, the owner decided to introduce the treatment. She received colostrum within the first 12 h of her life from the local high-quality colostrum reserves (>30% of sugar, digital Colostrum Refractometer, KRUUSE, Langeskov, Denmark). Supportive treatment with dexamethasone (0.05 mg/kg IV) and butafosfan with B12 vitamin (0.05 mg/kg IV) was also administered. During her first weeks of life, she was bottle-fed with a milk replacer and received supportive therapies including physiotherapy and assistance with standing up and learning to nurse whenever it became possible. The foal was isolated from other horses for a prolonged time due to its weakness and significantly smaller size.

**Figure 1:**
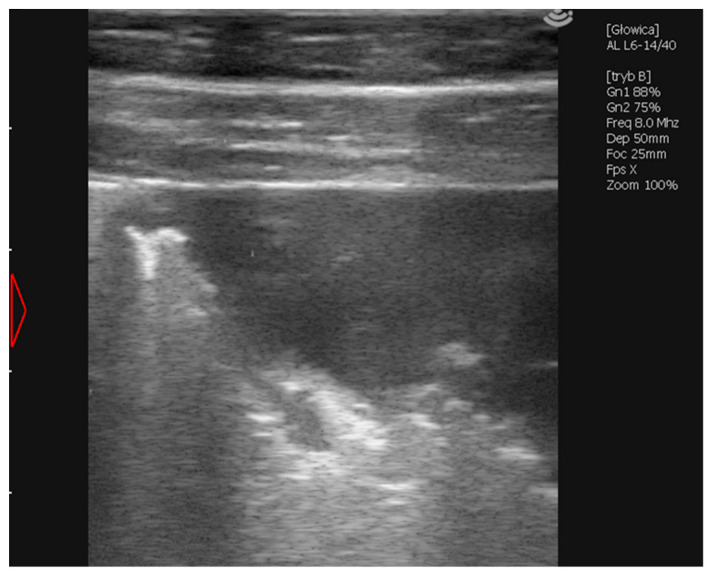
Ultrasonographic picture of an irregular abscessation observed in the right lobe from 9th ICS cranially during the first examination of Case 1. Visible parts of the abscess are 35.6 mm in width and 21.7 mm in depth.

Her first examination took place at 5 weeks of age. This stud participated in a lung ultrasound screening program; however, the inclusion of the filly was primarily delayed due to her isolation and specific maintenance. Physical examination revealed pyrexia (rectal temperature of 39.6 °C) with a mildly elevated respiratory rate (56 breaths/min), tachycardia (108 beats/min), capillary refill time (CRT) of 2 s, pink and moist mucus membranes and mandibular lymph nodes within a normal range. She had no audible abnormalities on the thoracic auscultation. During the ultrasound examination, multiple B- lines were detected bilaterally. A multifocal abscess of significant size was detected on the left side at the 12th intercostal space (ICS). Furthermore, a 10 mm abscess occurred at the 14th ICS on the right side, and an irregular abscessation was visible cranially from the 9th ICS (Figure 1). Antimicrobial treatment with clarithromycin (7.5 mg/kg 2/daily PO) and rifampicin (5 mg/kg 2/daily PO) was implemented on the same day and continued for the following ten weeks, according to the changes in the filly’s weight [1].

During the treatment, she presented both recurrent fever and lesions of variable size and number. In her third month of life, the filly developed clinical signs of retrobulbar abscess. It progressed to partial blindness and left-sided microphthalmia over the next month, despite the additionally implemented treatment of topical tropicamide and gentamicin.

The filly was monitored until she was nearly 5 months old. Neither clinical findings nor major ultrasound abnormalities were present at that time. She had a small number of B-lines of variable width, present mostly in the cranial parts of the lungs and suggestive of post-inflammatory fibrous changes.

The case came back in July 2020, when she was 14 months old. This yearling was appointed for an ultrasound examination due to her quickly deteriorating condition and significant dyspnoea. Noticeable underweight and left-side microphthalmia were observed. On physical examination, a rectal temperature of 37.9 °C with a mildly elevated respiratory effort (28 breaths/min) and heart rate (HR; 76 beats/min), CRT of 2 s, pink and moist mucus membranes and mandibular lymph nodes within a normal range were noted. Apparent and loud wheezes were detected during thoracic auscultation. No other clinical abnormalities were reported. Ultrasound examination revealed a significant amount of free fluid in the pleural cavity bilaterally, with multiple hyperechoic shadows suggestive of fibrin clots. A few days later the yearling died. A shortened field necropsy was performed on the same day. A significant amount of fibrotic, turbid pleural fluid was confirmed in the thoracic cavity. Moreover, the size of the lungs was severely decreased and macroscopically presented major post-inflammatory and fibrotic changes. A small amount of turbid, fibrotic fluid was also present in the abdominal cavity, with chronic inflammatory changes in intestinal mucosa and mildly enlarged mesenteric lymph nodes. The owner decided not to pursue further post-mortem diagnostics. Bacteriological examination of the lung parenchyma revealed the presence of *Rhodococcus equi* and *Streptococcus* sp. β-haemolytic type.

#### 2.1.2. Case 2—Problematic Bloodline

Two foals from the same Anglo-Arabian dam breeding line were undergoing a clinical and ultrasound examination as a part of a monitoring program aiming to recognize early signs of rhodococcosis in foals. Both mares, mother (Dam 1) and daughter (Dam 2) from this line had been successfully inseminated with two different, unrelated sires and were expecting foals in the 2020 breeding season. Both dams had a history of abortions or losses of foals in prior years; however, due to breeder change, it was not yet investigated.

#### 2.1.3. Case 2.1

##### Dam 2 Gave Birth to a Healthy Colt at the End of January 2020

This foal underwent his first monitoring examination at the 3 weeks of age. He had no history of previous health issues. On physical examination, a rectal temperature of 38.7 °C with a respiratory rate within normal and tachycardia (100 beats/min), CRT < 2 s, pale pink and moist mucus membranes and mandibular lymph nodes within a normal range were noted. No auscultation abnormalities were detected. Ultrasound examination revealed a small number of B-lines, which might be considered acceptable for a foal at his age. The monitoring was performed in two weeks intervals, with no significant exacerbations detected until 8 weeks of age.

The foal was reported pyrectic at 10 weeks. On physical examination, a rectal temperature of 40.1 °C with a mildly elevated respiratory rate and HR of 76 beats/min, CRT of 2 s, pale pink and moist mucus membranes, and mandibular lymph nodes within normal were noted. No auscultation abnormalities were detected. A small number of B-lines and a few small consolidations in the right cranial lobe were recognized during the ultrasound examination. Since then, the colt was treated with tulathromycin (2.5 mg/kg 1/week IM) and rifampicin (5 mg/kg 2/daily PO) according to the changes in the foal’s weight [1,13]. He was euthanized a month later, at 14 weeks old, due to progressive deterioration.

**Figure 2:**
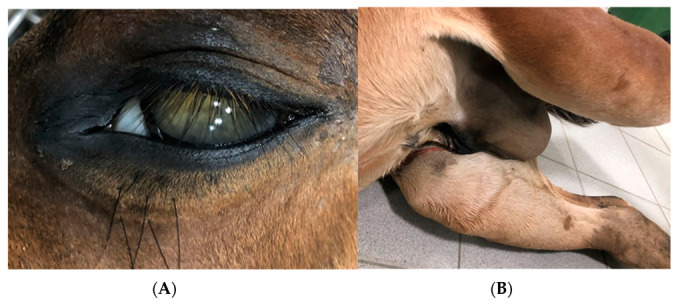
Anatomopathological findings concerning Case 2.1. Uveitis (**A**), scrotal edema and hernia (**B**).

**Figure 3:**
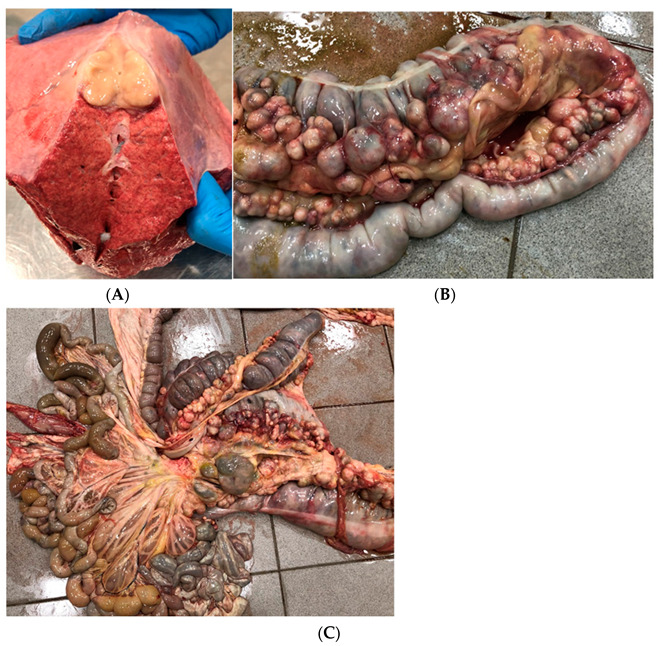
Anatomopathological findings concerning Case 2.1. Dissection of a pulmonary abscess (**A**), large intestine and mesenteric abscessation (**B**,**C**).

A necropsy was performed on the following day. The external evaluation showed significant underweight, bilateral uveitis (Figure 2a), and arthritis with a significant amount of synovial fluid, especially in the tarsal joints. Additionally, excessive overgrowth of the synovia was recognized. Internal evaluation of the thorax revealed fibrotic pleural fluid, inflammatory foci, and a few abscesses of 10–15 mm in diameter in the lung parenchyma (Figure 3a), particularly in the cranial and dorsal parts. Mediastinal lymph nodes were severely enlarged and purulent, which is rarely described [19]. Internal evaluation of the abdomen showed fibrotic, turbid fluid in the peritoneum, multiple abscesses along the large intestine with mesenteric lymph nodes abscessation (Figure 3b,c), and advanced inflammatory changes in the intestinal mucosa. Moreover, scrotal oedema (Figure 2b) with fibrotic, turbid fluid inside the tunica vaginalis, bilateral testicle atrophy, and inguinal hernia with omentum partially inside the scrotum were present. A bacteriological examination of the samples from the lung tissue and mesenteric lymph nodes confirmed the presence of *Rhodococcus equi*. The inflammatory changes in tarsal joints seemed to be immune-mediated aseptic polysynovitis since no microbiological culture from the synovial membrane samples was confirmed.

#### 2.1.4. Case 2.2

##### Dam 1 Gave Birth to a Healthy Filly at the Beginning of June 2020

This foal also underwent her first screening at 3 weeks old. She had no history of prior health issues. On physical examination, a rectal temperature of 38.6 °C with a respiratory rate within a normal range and HR of 104 beats/min, CRT < 2 s, pink and moist mucus membranes and mandibular lymph nodes within a normal range were detected. She had no abnormalities audible on thoracic auscultation. Ultrasound examination revealed a small number of B-lines, which might be considered acceptable for a foal of this age. During the next ultrasound examination (5 weeks old) clinical parameters of the filly stayed within a normal range; however, a significant number of B-lines up to 5 mm was recognized with a few small consolidations in cranial lobes.

**Figure 4:**
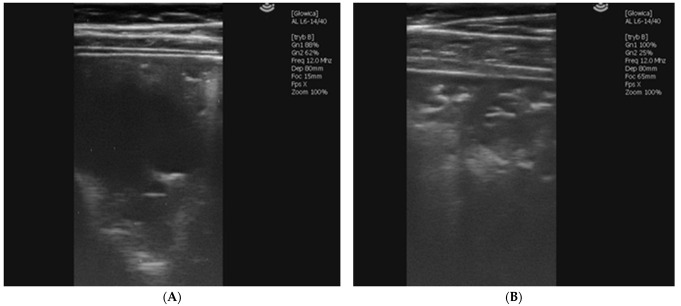
Ultrasonographic picture of an irregular abscessation with hypoechoic fluid in the left lobe in 11th ICS (**A**) and a multifocal abscessation in the right lobe from 11th ICS cranially in the right lobe (**B**) observed during the first examination of Case 2.2.

The filly was reported pyrectic with 39.9 °C being 7 weeks old. She also had a mildly elevated respiratory rate and tachycardia (96 beats/min), CRT of 2 s, pink mucus membranes, and mandibular lymph nodes within a normal range were detected. She had audible wheezes on thoracic auscultation. Ultrasound examination revealed two significant abscesses of 25 and 40 mm in diameter localized in 12 and 11 intercostal spaces, respectively on the left side (Figure 4a) and two abscesses on the right side, one of 43 mm in 11 ICS and another one, multifocal and irregular of significant size in 10–8 ICS (Figure 4b). The treatment was started on the same day (tulathromycin 2.5 mg/kg 1/week IM and rifampicin 5 mg/kg 2/daily PO) and conducted according to the changes in the filly’s weight [1,13]. The foal was examined for the last time at 12 weeks, with poor improvement and persistent recurrent fever, and died three days later.

A necropsy was performed on the following day. The foal was in moderate body condition. The examination revealed multiple variable sizes of abscesses and inflammatory changes in lung parenchyma and a moderate amount of turbid pleural fluid. No extrapulmonary changes were detected. Bacteriological examination of the lung parenchyma revealed the presence of *Rhodococcus equi* and *Streptococcus* sp. β-haemolytic type.

Due to significant health issues and deaths of the previous foals and suspicions of the breeder about congenital predispositions, it was decided to perform a laboratory test to determine the Immunoglobulin levels of the family. Accordingly, the mother’s (Dam 1), daughter’s (Dam 2), and grandson’s Ig levels were investigated in the laboratory at the beginning of May. The second examination took place in August and included Dam 1 and her filly. The results of the examinations are presented in Table 1.

The results indicated that none of the horses had IgM levels within a normal range, and all of the offspring showed significant IgM deficiency. Thus, it might be suspected that Dam 1 might pass some congenital immunological defect affecting the foals’ losses. No apparent clinical signs that might indicate infection were detected in both mares during the blood sampling. As a result, the breeder decided to withdraw both mares from reproduction without further investigation.

#### 2.1.5. Case 3—Suspicion of Extrapulmonary Disorders

A 6-week-old Arabian filly was appointed for a clinical and ultrasound examination due to weakness, mild diarrhoea, reduced appetite, no weight gain, and apathy for a few days. She was not included in an ultrasound screening program. Her daily rectal temperature measurements had stayed within a normal range and there have been no other health complaints about her since birth.

During the first physical examination, a rectal temperature of 38.3 °C with a respiratory rate within a normal range and HR of 64 beats/min, CRT of 2 s, pink and moist mucus membranes and mandibular lymph nodes within normal were detected. She had mild auscultation wheezes present on the left side of the thorax. A small number of B- lines were detected bilaterally during the ultrasound examination, which may be considered normal for a foal at this age. However, on the left side at the 16th intercostal space, an abscess of 45 mm was detected. This location is considered less common for a single pulmonary consolidation since most of the lesions are usually detected in the cranial and medial parts of the lungs [20]. Moreover, her clinical appearance described above implied the possibility of extrapulmonary disorders connected with abdominal abscessation; however, no such changes were available to visualize during the ultrasound examination. Due to the significant size of the abscess and the poor general condition of the filly, antimicrobial treatment was recommended and implemented on the following day. She was treated with tulathromycin (2.5 mg/kg 1/week IM) according to the changes in the filly’s weight [13]. The antimicrobial treatment was conducted until she reached 11 weeks when no signs of abscessation were detected during the ultrasound examination.

Clinical parameters stayed within a normal range during the treatment, and no fever incidents were detected. It took approximately two weeks for the foal to regain the expected condition and the abscess to reduce in size (26.4 mm). On the following examination, the abscess became irregular due to fibrotic tissue appearing in the lung parenchyma and the exact abscess’ size was not available to be measured. During the last examination, there were no remains of the abscess detected.

#### 2.1.6. Case 4—Drug-Induced Hyperthermia

A 5-week-old Angloarabian filly, was appointed for a clinical and ultrasound examination due to pyrexia and dyspnoea. The haematologic results from the day of pyrexia onset presented in Table 2 indicate acute inflammation and dehydration. Her daily rectal temperature measurements stayed within a normal range until two days before the ultrasound examination, when it reached 39.2 °C. She was given benzylpenicillin (8 mg/kg, IM) with streptomycin (10 mg/kg, IM) and metamizole (22 mg/kg, IV) for three days which reduced the fever.

During the first examination two days later, she presented dyspnea. Her rectal temperature was 38.6 °C HR 64 beats/min, CRT 2 s, pink mucus membranes, mandibular lymph nodes within a normal range, and she had no other apparent health issues. She had no audible auscultation abnormalities on any side of the thorax. A moderate number of B- lines were detected bilaterally during the ultrasound examination, which might be considered acceptable for a foal at this age for a short period. An irregular abscess of significant diameter was detected on the left side at the 8–6th intercostal space. On the right side of the thorax, another irregular abscess occurred at the 11–8th intercostal space. Antimicrobial treatment was recommended and implemented on the same day. The filly was treated with clarithromycin (7.5 mg/kg 2/daily PO) and rifampicin (5 mg/kg 2/daily PO) according to the changes in the filly’s weight [1]. The antimicrobial treatment was conducted until the filly reached 14 weeks when no abnormalities were detected during an ultrasound examination.

**Figure 5:**
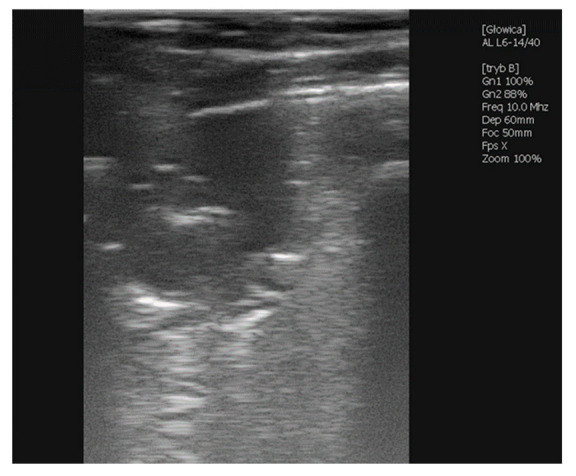
Ultrasonographic picture of a circular abscessation observed in the left lung, 16th ICS during the second examination of Case 3. Visible parts of the abscess are 26.4 mm in depth and 23.3 mm in width.

The second examination of the filly happened to occur at the beginning of June (7 weeks of age). The outdoor temperatures were reaching 30 °C during the daytime and the foal presented rectal temperatures from 39.5 °C up to 41.0 °C for a few days, with little response to antipyretic drugs. However, an ultrasound examination revealed a significant reduction in the abscesses’ size (Figure 5). Since macrolides are known to have an anhidrosis potential in some horses, such a case was suspected. Therefore, the stable staff was asked to wet the filly with cold water regularly and to carefully monitor the rectal temperatures for a few days. Hyperthermia secondary to macrolide-induced anhidrosis seemed to be the proper diagnosis and owing to regularly wetting the filly and careful temperature checks, no other antipyretic drugs were needed. The procedure was implemented throughout the treatment, which took nearly two months in total. Wetting was conducted whenever the outdoor temperature was high and the stable staff reported increased rectal temperature (above 39 °C) during the morning examination.

## 3. Discussion

*Rhodococcus equi* is commonly associated with respiratory signs in foals from endemic studs. However, attaining reports about extrapulmonary findings and persisting impact of *Rhodococcus* on the health of endemic areas may indicate underlying issues. Extrapulmonary findings are often bound with more severe cases and worse prognoses [6,8,25,26,27]. This approach may be very accurate, but our ability to diagnose some of them, especially those connected with abdominal abscessations, is limited due to the most common locations of the changes, often superimposed by gaseous loops of the intestines. Moreover, clinical findings of abdominal abscessation are usually very unspecific and often restricted to diarrhoea and underweight. Those cases can also be difficult to differentiate from macrolide-induced problems. Many extrapulmonary incidents are confirmed and reported only based on post-mortem examination [25,27], or, in the case of skeletal abscesses during the radiological examination due to different reasons. Both types of changes may be significantly underdiagnosed. In the aforementioned Case 2.2, abdominal signs were suspected due to cachexia, persisting diarrhoea and slow response to antimicrobial treatment.

Ophthalmic changes are barely reported and generally worsen prognoses [6,26]. What is more, the papers describe cases of rhodococcal infections referred for hospital treatment, which are usually more severe [6]. In the alluded cases, ophthalmic changes terminated in the loss of foals. Filly described in Case 1 seemed to have the retrobulbar abscess at least partially cured. The eye bulb became microphthalmic, but the filly reacted properly to rapid movements and light changes on the affected side.

One of the most important problems while managing rhodococcosis is undoubtedly an extremely limited number of therapeutics effective in vivo [1,4,12,13,14,25,28]. Additionally, increasing antimicrobial resistance presents a serious threat to both human and veterinary medicine [29,30,31]. Unfortunately, in many countries, we lack such data, or, as in Poland, they are limited only to some regions [28,31]. In all of the aforementioned fatal cases, antimicrobial resistance against antibiotics selected for treatments was not detected.

Antimicrobial resistance does not end the list of disadvantages. Digestive issues and dysbiosis resulting from the use of certain drugs, especially macrolides, may create a serious risk for both mares and foals [32]. What is more, macrolide-induced anhidrosis may present even greater peril. These reactions in certain foals are connected with ion channels and β_2_-adrenergic receptors, but the proceedings and prognosis in such incidents are still not settled [33,34]. Up to date, in case of such antimicrobial adverse effects, the only solution implemented in cooperating studs relies on careful monitoring of the foals by appropriately qualified and responsible stable staff and their minute fulfilment of the recommendations.

Genetic susceptibility for rhodococcal infections in foals was not established and scientific reports on that matter are also extremely limited [2,35]. Some genes were suspected to possibly influence the development and course of clinical rhodococcosis but were not yet extended for more biological samples and results. Other papers claim no strict reliance on genetic factors in the development of clinical rhodococcosis [35]. It might be possible that some mares deliver foals that are of higher risk due to lower colostrum quality. Some foals may also be more prone to the disease due to increased exposure to pathogens caused by environmental factors or higher periodic faecal shedding among the group [3,4,36,37,38] but not for any strictly genetic problem. Thus some epigenetic and environmental factors may become crucial for further investigation.

Another suspicion arises around recurrent problems in certain bloodlines, frequently reported by field practitioners but not mentioned in scientific reports. In such cases, stud veterinarians often briefly investigate the matter in local laboratories; however, it usually ends with no clear answer. The key to resolving the problem may stay beyond genetic profile. For instance, some authors report a correlation between early Equine Herpes Virus type 1 or 2 (EHV1 and 2) infections and an increased risk of developing clinical rhodococcosis [39,40]. This hypothesis may be at least partially true for many reasons. The possibility of post-viral immunosuppression after early herpes infection being connected with increased susceptibility to clinical rhodococcosis is worth considering. Other species, including humans, significantly less affected by environmental *Rhodococcus,* suffer from the disease mostly when immunologically deficient [4,10,41,42,43,44,45]. What is more, susceptibility often occurs simultaneously with the highest exposure to a significant number of clinical and subclinical foals confirmed to have increased faecal shedding [36]. This hypothesis can also be supported considering that clinical signs usually develop during the immunological gap in foals. Nevertheless, these matters are still to be investigated.

The deficiency in immunoglobulins type M (IgM) of possibly inherited background suspected above partially binds earlier hypotheses. Reports about deficient IgM in horses are extremely rare. Primary selective IgM deficiency is usually mentioned in the manuals with a grave prognosis due to the foals being highly prone to multiple and recurrent bacterial and viral infections, especially pneumonia. The issue typically occurs in Arabians or AQH breeds [24,46]. The IgM deficiency was also described in a colt with a confirmed impaired B-cell response. IgMs are known to take part in initial antigen recognition and humoral activation, so this may partially explain the primary inability to fight infection in such foals and the following deterioration [47]. Secondary selective IgM deficiency is associated with lymphoma in adult horses; however, despite high sensitivity, it failed to be its only marker during diagnostic trials [48,49]. Although IgG seems to play a major role in clearing rhodococcal infections, IgM levels were also found elevated in the bronchoalveolar lavage (BAL) of foals affected with rhodococcosis [49]. These immunologic patterns remain yet undetermined; however, congenital immunological deficiencies could be considered in some recurrent problems of certain bloodlines.

The study presents certain limitations. The monitoring program was introduced as an additional tool to improve diagnostic procedures and treatment outcomes in endemic studs, thus the veterinarian performing lung ultrasound diagnosis was only an advisor to the stud veterinarians, who held full responsibility for the decisions regarding diagnosis and treatment. Moreover, the article gathers a few retrospective clinical cases, which unfortunately made most of the additional diagnostic procedures no longer available to perform.

## 4. Conclusions

Regardless of being an old and common dilemma, *Rhodococcus equi* and its clinical presentation remain a very challenging problem in endemic horse studs. What is particularly important is to preserve both scientific and clinical points of view to critically assess the ideas and implement proper solutions in managing the disease in field conditions, which remains an ultimate goal in solving clinical problems.

## Figures and Tables

**Table 1:** Results of immunoglobulin levels investigation. The Ig reference values for foals above 60 days of life are the same as for adult horses.

Horse	Results from the Beginning of May 2020		Results from the End of August 2020		
	IgG (Ref. Value 500–2000)	IgM (Ref. Value 90–150)	IgG (Ref. Value 500–2000)	IgM (Ref. Value 90–150)	IgA (Ref. Value 60–130)
Dam 1	1480 mg/dL	340.1 mg/dL	2190 mg/dL	272.3 mg/dL	55.4 mg/dL
Filly from Dam 1			630 mg/dL	4.5 mg/dL	101.9 mg/dL
Dam 2 *	1310 mg/dL	34.8 mg/dL			
Colt from Dam 2	1390 mg/dL	14.2 mg/dL			

* Daughter of Dam 1.

**Table 2:** Haematological results of Case 4 from the day of pyrexia onset with reference values [24].

	RBC	MCV	HCT	PLT	MPV	WBC	HGB	MCH	MCHC	LYMF	GRAN	MID
**Ref. value**	6.5–9.99	38–53	33–48	115–450	-	5.0–12.6	11–16	12–16	31–37	1.4–2.3	2.75–8.19	-
**Case 4**	9.96	32	31.9	360	7.3	29.7	20.7	20.8	64.8	4.6	23.5	1.5

RBC—red blood cell count; MCV—mean cell volume; HCT—haematocrit; PLT—platelet count; MPV—mean platelet volume; WBC—white blood cell count; HGB—haemoglobin; MCH—mean cell haemoglobin; MCHC—mean corpuscular haemoglobin concentration; LYMF—lymphocytes; GRAN—granulocytes; MID—mid-range absolute cell count.

## Data Availability

Not applicable.
